# Deciphering the Genome-Wide Transcriptomic Changes during Interactions of Resistant and Susceptible Genotypes of American Elm with *Ophiostoma novo-ulmi*

**DOI:** 10.3390/jof8020120

**Published:** 2022-01-26

**Authors:** Md Tabibul Islam, Jose Freixas Coutin, Mukund Shukla, Amandeep Kaur Dhaliwal, Martha Nigg, Louis Bernier, Sherif M. Sherif, Praveen K. Saxena

**Affiliations:** 1 Alson H. Smith Jr. Agricultural Research and Extension Center, School of Plant and Environmental Sciences, Virginia Tech, Winchester, VA 22602, USA; tabibul@vt.edu; 2 Department of Plant Agriculture, Gosling Research Institute for Plant Preservation (GRIPP), University of Guelph, Guelph, ON N1G 2W1, Canada; joeph84@gmail.com (J.F.C.); mshukla@uoguelph.ca (M.S.); amnsept@gmail.com (A.K.D.); 3 Centre d’Étude de la Forêt, Université Laval, Québec, QC G1V 0A6, Canada; martha.nigg@gmail.com (M.N.); Louis.Bernier@sbf.ulaval.ca (L.B.)

**Keywords:** American elm, Dutch elm disease, *Ophiostoma novo-ulmi*, RNA-Seq, defense mechanism

## Abstract

Dutch elm disease (DED), caused by *Ophiostoma novo-ulmi* (*Onu*), is a destructive disease of American elm (*Ulmus americana* L.). The molecular mechanisms of resistance and susceptibility against DED in American elm are still largely uncharacterized. In the present study, we performed a *de novo* transcriptome (RNA-sequencing; RNA-Seq) assembly of *U. americana* and compared the gene expression in a resistant genotype, ’Valley Forge’, and a susceptible (S) elm genotype at 0 and 96 h post-inoculation of *Onu*. A total of 85,863 non-redundant unigenes were identified. Compared to the previously characterized *U. minor* transcriptome, *U. americana* has 35,290 similar and 55,499 unique genes. The transcriptomic variations between ‘Valley Forge’ and ‘S’ were found primarily in the photosynthesis and primary metabolism, which were highly upregulated in the susceptible genotype irrespective of the *Onu* inoculation. The resistance to DED was associated with the activation of RPM1-mediated effector-triggered immunity that was demonstrated by the upregulation of genes involved in the phenylpropanoids biosynthesis and PR genes. The most significantly enriched gene ontology (GO) terms in response to *Onu* were response to stimulus (GO:0006950), response to stress (GO:0050896), and secondary metabolic process (GO:0008152) in both genotypes. However, only in the resistant genotype, the defense response (GO:0006952) was among the topmost significantly enriched GO terms. Our findings revealed the molecular regulations of DED resistance and susceptibility and provide a platform for marker-assisted breeding of resistant American elm genotypes.

## 1. Introduction

*Ophiostoma* species (i.e., *Ophiostoma novo-ulmi* and *Ophiostoma ulmi*) are the fungal pathogens causing Dutch elm disease (DED), which is a lethal vascular wilt disease of elms worldwide including American elm (*Ulmus americana*) [1,2]. *O. ulmi* caused the original DED epidemic in Europe and North America in the mid-1900s. During the second half of the 20th century, the more aggressive *O. novo-ulmi (Onu)* largely replaced *O. ulmi* and is responsible for the ongoing DED pandemic [3]. In North America, overland spread of DED from infected to healthy elms is facilitated by the native elm bark beetle (*Hylurgopinus rufipes*) and its European counterpart (*Scolytus multistriatus*), and the symptoms first appear on upper crown branches as wilting and yellowing leaves [4,5,6]. Generally, soon after one branch becomes symptomatic, adjacent branches also show symptoms, which is followed by significant crown dieback. When the bark is peeled off infected branches, longitudinal, brown-colored streaks in the outer rings of the sapwood are visible, and it is the most distinctive symptom of DED observed in the field. These fungi spread within stems and roots of living elms both by passive transport of spores and by the mycelial growth of colonies initiated by spores that germinate in the xylem. Dutch elm disease fungi can also infect healthy elms adjacent to a diseased elm through root grafts [7,8].

*Ophiostoma* species causing DED grow and reproduce within elms and are generally considered hemibiotrophic fungal pathogens [9]. However, these pathogens can be biotrophic sometimes, feeding on living tissues of the elm tree, and at other times, necrotrophic, getting nutrition from dead elm tissue [9]. Long-term control and management of DED primarily rely on the identification and development of resistant elm genotypes [10,11]. Since the disease appeared, several breeding programs have employed Asian elms such as *U. pumila* or *U. wallichiana* as the source of resistance, as they have been shown to be less susceptible to DED. The genetic heritability of resistance has been shown by crossing native and Asian elms [12,13] and crossing susceptible and resistant Iberian *U. minor* genotypes [14]. The transcriptomic changes of Iberian *U. minor* genotypes with contrasting resistance to DED showed that the phenylpropanoids biosynthesis pathway played a central role in the tolerance mechanism against DED [15]. Similarly, the transcriptomic analysis of *U. americana* calli inoculated with *Onu* showed that the elm transcripts encoding the enzymes involved in the phenylpropanoids metabolism and pathogenesis-related (PR) proteins were enhanced and hence signify the role of phenolics and PR proteins in the DED resistance mechanism [16]. The induction of defense-related genes and plant hormone jasmonic acid (JA) during the necrotrophic phase of DED has also been characterized as a resistance mechanism by comparing resistant and susceptible *U. americana* genotypes. Furthermore, at the early stage of DED, the application of salicylic acid (SA) enhanced elm resistance in the field and indicated that the coordinated action of SA and JA might be crucial in DED, as reported for resistance to diseases caused by other hemibiotrophic pathogens [9,17,18]. Despite these efforts toward dissecting the defense mechanisms against DED, the molecular responses of resistant and susceptible American elm genotypes to DED at the whole-transcriptmic level are still unknown.

Genome-wide transcriptome analysis has widely been used in several plant pathosystems as an efficient method for elucidating the molecular and genetic mechanisms involved in disease resistance [19,20]. Advances in understanding plant–microbe interactions have been enabled by the availability of plant genome sequences and the development of associated bioinformatics tools and resources [15,19,20]. Comparative transcriptional analysis, using RNA-sequencing (RNA-Seq), is a prominent approach for identifying genes that are differentially expressed between two contrasting treatments/genotypes. However, a comprehensive study of the genes involved in molecular mechanisms of resistance in *U. americana* has not been performed thus far. The existing variation in *U. americana* genotypes for resistance and susceptibility traits can be explored by RNA sequencing. This knowledge is indispensable for developing resistant genotypes through breeding to preserve the threatened American elm.

The cultivar ‘Valley Forge’ is considered relatively more resistant to DED than other cultivars such as ‘Princeton’, ‘Delaware’, and ‘New Harmony’ [21]. The induction kinetics of genes after infection with *O. novo-ulmi* (*Onu*; MH75-4O) were examined in resistant (Valley Forge) and susceptible genotypes, and indicated that the differences among genotypes are due to timing and level of the gene expression rather than the presence or absence of disease-responsive genes [9]. At 96 h post-inoculation (hpi), genes encoding pathogenesis-related proteins showed ≥2-fold higher expression in ‘Valley Forge’ than the susceptible clone along with abundant growth of conidia and hyphae in the susceptible clone [9]. In the present study, we used Illumina HiSeq 2500 paired-end sequencing followed by a de novo transcriptomes assembly to reveal elm transcriptomic changes in response to *Onu.* Two contrasting genotypes, including the resistant Valley Forge (V) and a susceptible clone (S), were exposed to *Onu*, and samples were collected at 0 hpi and 96 hpi. The objective of the work reported herein was to identify genes upregulated and downregulated in response to *Onu* infection and provide a platform for characterizing them further for developing resistance in American elm against DED. Fungal transcriptomes were also recovered and analyzed; results will be presented in a separate contribution.

## 2. Materials and Methods

### 2.1. Experimental Materials, Fungal Isolate, and Inoculation Conditions

All plant material, inoculum (*O. novo-ulmi*; MH75-4O), inoculation conditions, and tissue collection were described previously [9]. Briefly, four-year-old American elm ‘Valley Forge’ was used as a line with high degree of resistance to DED. The elm genotype highly susceptible to DED was selected from in vitro elm germplasm collection at the Gosling Research Institute for Plant Preservation (GRIPP), University of Guelph, Ontario, Canada. The clones were kept under 16 h light at 24 °C and 8 h darkness at 20 °C, and light intensity was set at 110 μmolm^−2^ s^−1^ (LI-250 A, LI-COR; Biosciences, Lincoln, NE, USA) under greenhouse conditions and later moved outside for the experiment. Both clones were inoculated with *O. novo-ulmi* (*Onu*) along the main stem, and stem segments 2 cm^2^ around each inoculation point were collected at 0 and 96 hpi for transcriptome profiling. To prepare the *Onu* inoculum, yeast spore suspensions were prepared as described elsewhere [9], and the spore density was adjusted to 10^7^ spores/mL based on initial densities determined with a hemocytometer. The highly virulent and sequenced strain *O. novo-ulmi* ssp *novo-ulmi* H327 could not be used in this study because of the quarantine regulations in Ontario [22]. For RNA sequencing, three biological replicates were collected for resistant and susceptible genotypes at 0 and 96 hpi, resulting in 12 libraries. The inoculated plants were assessed for the disease incidence and severity at 60 days post-inoculation (dpi).

### 2.2. RNA Extraction and Sequencing

The detailed protocols for RNA extraction, cDNA synthesis, and quantitative real-time PCR were mentioned in the previous study [9]. Briefly, the CTAB protocol was used for RNA extraction, which is followed by purification with a RNeasy Mini Kit (Qiagen). The cDNA was synthesized using a Superscript VILO cDNA Synthesis kit (Invitrogen, Burlington, ON, Canada). The cDNA libraries were prepared and sequenced using paired-end reads on the Illumina HiSeq 2500 platform.

### 2.3. Ulmus Americana Transcriptome Analysis

The filtered reads were obtained after removing adapter sequences. The assembled *U. americana* putative scaffolds were filtered by size (≥300 bp) and annotated using the non-redundant protein database downloaded from NCBI (ftp://ftp.ncbi.nlm.nih.gov/blast/db/, accessed on 1 March 2018) in BLASTx v. 2.8.0 software. Then, filtered reads were mapped to the assembled *Ulmus americana* transcriptome for quantification purposes using BWA v. 0.7.4-r385 [23]. Afterwards, read counts were assessed in R software [24] for differential gene expression analysis using the package *edgeR*. Within *edgeR*, the function *plotMDS* was implemented to assess for variability among biological replicates in a bi-dimensional scaling plot in which distances correspond to leading log-fold changes between each pair of RNAseq samples. The *glmTreat* function was employed for a rigorous differential gene expression analysis based on the variability observed among biological replicates. Significantly differentially expressed genes (DEGs) were identified with log2FC > 2 and adjusted *p*-value < 0.05 as the upregulated DEGs, and log2FC < −2 and adjusted *p*-value 0.05 as downregulated DEGs. Volcano plots of DEGs were generated using MetaboAnalyst (http://www.metaboanalyst.ca, accessed on 26 November 2021).

### 2.4. Comparative Analysis between U. americana and U. minor Transcriptomes

The FASTA file corresponding to the *U. minor* transcriptome, accession number SRR1687227 was downloaded from NCBI Short Read Archive. Thereafter, the FASTA files corresponding to *U. minor* and *U. americana* transcriptomes were converted to two local databases using the *makeblastdb* function of BLAST v.2.8.0 software. The *blastn* application was used to compare the FASTA files corresponding to each *Ulmus* transcriptome against their local database counterpart. Shared genes between *U. minor* and *U. americana* were filtered from the original FASTA files, and the remaining unique genes were compared against the non-redundant protein database downloaded from NCBI (ftp://ftp.ncbi.nlm.nih.gov/blast/db/, accessed on 1 March 2018) using BLASTx v.2.8.0. The unique genes having E-values ≤ 1 × 10^−6^ and similarity ≥ 70% were further studied to identify the top 10 species with the highest number of hits produced by BLASTx. Unique contaminant genes unrelated to plant species and potential artifacts were identified and removed using the package *taxonomizr* in R.

A transcriptome comparison was performed using the FASTA files of *U. minor* and *U. americana* transcriptomes in order to determine and annotate unique genes for each species. To this end, the FASTA files for each transcriptome were converted to two local databases (DB), which were used as templates for BLASTX software (*U. minor* FASTA file vs. *U. americana* local DB, and vice versa).

### 2.5. Gene Ontology (GO) and Kyoto Encyclopedia of Genes and Genomes (KEGG) Pathways Enrichment Analysis

A BLASTx-based approach was run from the command prompt to extract the GenInfo Identifier (GI) numbers from the best BLASTx hits with the *Ulmus americana* transcriptome, having E-value ≤ 1 × 10^−6^and similarity ≥ 70%. The *Arabidopsis* genes corresponding to American elm genes were obtained as the best hit of *Arabidopsis.* The Significant Differentially Expressed Genes (DEGs) were annotated with gene ontology (GO) by AgriGO v2.0 [25] using *Arabidopsis* TAIR10 genomes. The KEGG pathway enrichment analysis of DEGs was conducted using the web-based DAVID v6.8 tool (https://david-d.ncifcrf.gov/, accessed on 11 December 2021). The hypergeometric statistical model and *p* values were adjusted by the Benjamini and Hochberg method. The GO terms and KEGG pathways with FDR < 0.05 were regarded as significantly enriched. The GO enrichment networks were analyzed by BiNGO in Cytoscape [26].

### 2.6. Validation of Candidate Genes Expression by Quantitative Real-Time PCR

Total RNA isolated as described previously [9] was treated with DNase I, and first-strand cDNA was synthesized from 2.5 μg of DNase treated RNA using the SuperScript^®^ VILO™ cDNA Synthesis Kit (Invitrogen, Burlington, ON, Canada). A 2.5 µL aliquot representing a 20-fold dilution of the cDNA was used as a template for qRT-PCR using gene-specific primers (Table S1). All treatment samples were assayed in duplicate in a 10 µL reaction containing Bio-Rad SYBR Green and 5 pmol of each primer on a CFX Connect Real-Time PCR Detection System (Bio-Rad, Mississauga, ON, Canada). The cycle threshold (Ct) value of each candidate gene was compared to the corresponding reference gene, *Splicing factor 3B-F*, and 0 h post-inoculation (hpi) in susceptible (S) genotype as treatment control and relative expression values were calculated according to the 2^−ΔΔCt^ method [27]. Results were statistically analyzed using the CFX manager software (Bio-Rad). The RNA used for qRT-PCR investigations was extracted from the same tissues used for transcriptome analysis.

## 3. Results

### 3.1. Development of Dutch Elm Disease (DED) Symptoms

**Figure 1:**
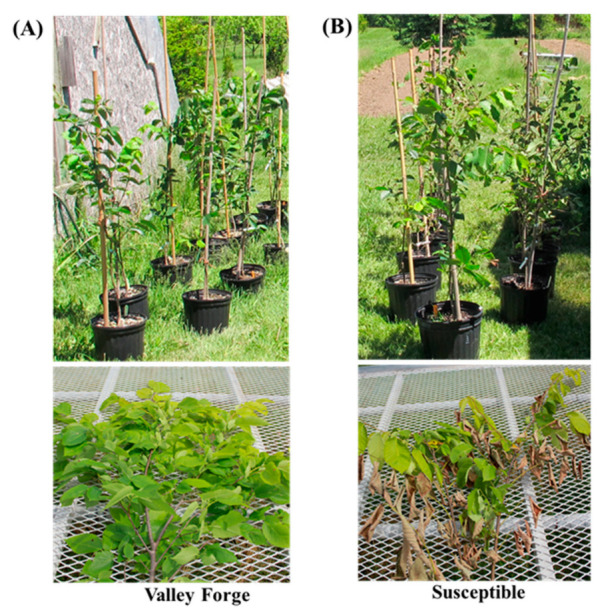
Development of Dutch elm disease symptom in American elm genotypes at 60 days post-inoculation with *Ophiostoma novo-ulmi.* (**A**) Resistant ‘Valley Forge’; and (**B**) susceptible clones.

In response to *Onu*, the American elm cultivar ‘Valley Forge’ was found almost symptomless (Figure 1A), whereas the susceptible genotype showed severe DED symptoms at 60 dpi (Figure 1B). Internal DED symptoms appeared as brown and dark brown streaks right under the bark of the susceptible genotype.

### 3.2. Analysis of the American Elm Transcriptomes

A transcriptomic analysis using the RNA-Seq technology was performed to characterize the molecular defense responses in American elm saplings against *O. novo-ulmi* (*Onu*). For maximal read depth, all RNA samples were depleted of the highly abundant rRNA, which strongly interferes with the sequencing reactions in Illumina platforms [28,29]. The Illumina HiSeq 2500 platform was used to generate paired-end reads for 12 cDNA libraries representing three biological replicates of two American elm genotypes; DED-resistant ‘Valley Forge’ and DED-susceptible (herein referred to as Susceptible, S) at 0 and 96 h post-inoculation with *O. novo-ulmi* strain MH75-4 O. For libraries of both American elm genotypes at each post-inoculation time point, the average number of raw sequence reads ranged from 66.4 to 75.8 million (Table 1). Among the total mapped reads, less than 0.22% aligned to more than one location in the reference genome, while the remaining resulted in single mappings. The assembly revealed that approximately 70% of the unmapped reads, representing an average of 50.8 million raw reads, were de novo assembled into *U. americana* transcriptome.

### 3.3. Comparative Transcriptome Analysis

BLASTX analyses aligned approximately 53.5% of the *U. minor* unigenes to *U. americana* local DB (*n* = 85,863; E-value ≤ 1 × 10^−6^; similarity ≥ 70%) and 41.1% of *U. americana* unigenes to *U. minor* local DB (*n* = 73,917; E-value ≤ 1 × 10^−6^; similarity ≥ 70%).

**Figure 2:**
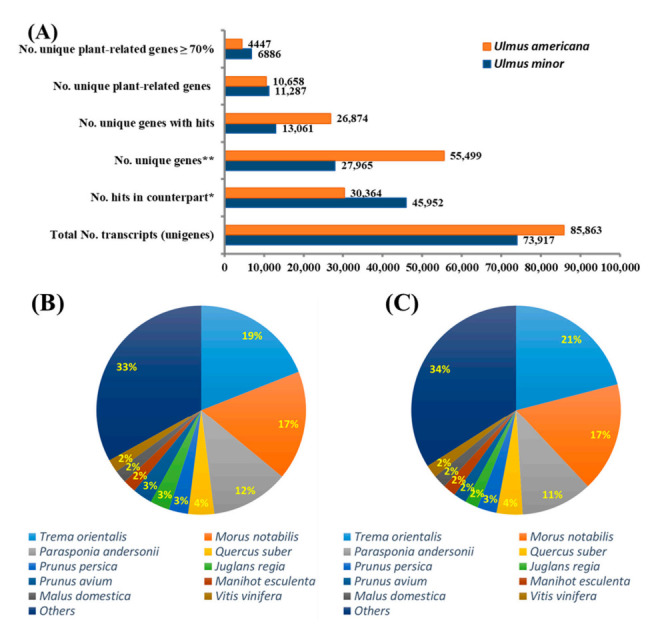
(**A**) Comparative transcriptome analysis between *Ulmus minor* and *U. americana*. Blastn v.8.0 was used to compare the transcriptome of both *Ulmus* species. All common genes between the *Ulmus* spp. showed a percentage of identity higher than 70%. Unique genes that returned no hits between the *Ulmus* transcriptomes were compared against the non-redundant protein database using a BLASTX approach. (**B**) The number of hits resulting from a BLASTX analysis of unique plant-related genes from *U. minor* transcriptome against the non-redundant protein database. (**C**) The number of hits resulting from a BLASTX analysis of unique plant-related genes from *U. americana*. * Refers to common genes between *U. americana* and *U. minor*. ** Refers to unique genes for each of the two *Ulmus* sp. transcriptomes that are not similar between them.

Although the similarity cut-off was set at 70%, the majority (91.3%) of common genes between *U. minor* and *U. americana* transcriptomes showed similarities higher than 90%. In addition, using the BLASTX software, the unique genes of each transcriptome were compared to the non-redundant protein database downloaded from NCBI, annotating approximately 61% genes from *U. minor* and 42% genes from *U. americana* as plant-related transcripts (Figure 2A). The number of hits per plant species in the non-redundant protein database for each *Ulmus* sp. transcriptome was generated using the BLASTX-based approach. As shown in (Figure 2B,C) for each *Ulmus* transcriptome, nearly 70% of the best hits produced by BLASTX were concentrated in 10 species. The remaining 30% resulted from 118 and 233 species for *U. minor* and *U. americana* transcriptomes, respectively. The top 5 best-hit species for *U. minor* and *U. americana* transcriptomes were all classified as Angiosperms within the order Rosales. This suggests that most of the unique genes for *U. minor* and *U. americana*, regardless of having similarities lower than 70%, could still have related functions, as these were similar to other transcripts from the same plant species.

### 3.4. Differential Gene Expression Analysis

**Figure 3:**
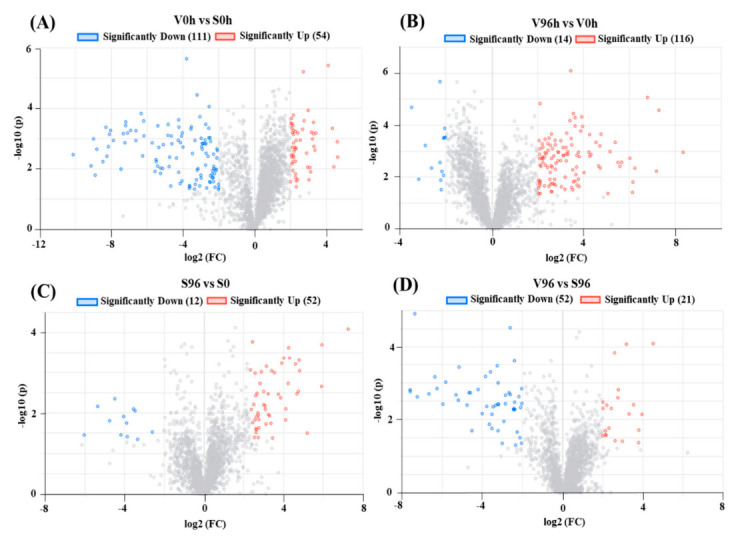
Volcano plots of differentially expressed genes (DEGs). The X-axis shows the log2 FC in gene expression, (**A**) between non-inoculated ‘Valley Forge’ (V) and ‘Susceptible’ (S) American elm; (**B**) between 0 and 96 hpi in ‘Valley Forge’ (V) American elm; (**C**) between 0 and 96 hpi in ‘Susceptible’ (S) American elm; and (**D**) between Valley Forge’ (V) and ‘Susceptible’ American elm at 96 hpi. The Y-axis shows the statistical significance of the differences. Splashes represent different genes. FC: Fold change; hpi: h post-inoculation; −log10(p): the corrected *p*-value.

A total of 5329 genes were differentially expressed between Valley Forge (V) and Susceptible (S) American elm genotypes in this study. Significant DEGs (upregulated; log2FC > 2 and adjusted *p*-value < 0.05, and downregulated; log2FC < −2 and adjusted *p*-value < 0.05) are presented in volcano plots (Figure 3A–D). A total of 165, 130, 64, and 73 significant DEGs were identified from the comparisons between V0h and S0h, V96h and V0h, S96h and S0h, as well as V96h and S96h, respectively (Figure 3).

### 3.5. GO Enrichment Analysis

Gene ontology (GO) enrichment was performed using AgriGO v2.0 [25] to generate an overview of the functional classifications of the DEGs associated with *U. americana* responses to *Onu*. The DEGs between the ‘Valley Forge’ and ‘Susceptible’ American elm genotypes yielded the most significant GO terms regardless of the pathogen inoculations. These GOs were involved in the metabolic process, cellular process, and primary metabolic process (Table 2). On the other hand, in responses to *Onu*, the most significantly enriched GO terms were the response to stimulus (GO:0050896), response to stress (GO:0006950), and secondary metabolic process (GO:0019748) in both resistant and susceptible genotypes. Interestingly, the defense response (GO:0006952) was the topmost significantly enriched GO term in the resistant genotype exposed to *Onu.*

### 3.6. Pathway Enrichment Analysis

Significantly DEGs were analyzed for pathway enrichment using the Kyoto Encyclopedia of Genes and Genomes (KEGG) pathways. The photosynthesis (ko00195), carbon metabolism (ko01200), and citrate cycle (ko00020) were among the highly enriched pathway between the resistant and susceptible elm genotypes irrespective of *Onu* inoculation. The secondary metabolism, mainly the phenylpropanoids biosynthesis (ko00940), was highly annotated in response to *Onu* infection (Table 3).

### 3.7. GO Network Analysis

**Figure 4:**
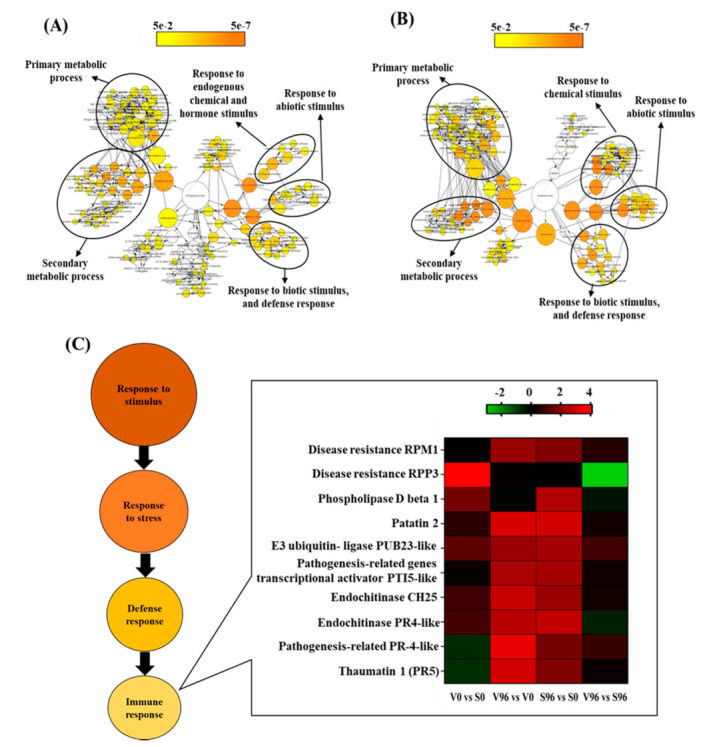
GO network visualization for the significant DEGs in (**A**) resistant (V96h vs. V0h) and (**B**) susceptible (S96h vs. S0h) interactions between *Ulmus americana* and *Ophiostoma* *novo-ulmi*. (**C**) Expression pattern of the immune-responsive genes in American elm.

DEGs in resistant (V96h vs. V0h; Figure 4A) and susceptible (S96h vs. S0h; Figure 4B) American elm genotypes in response to *Onu* were associated with three GO terms: response to stimulus (chemical, abiotic, and biotic), metabolic process (secondary and primary metabolism), and responses to stress (defense response, immune response). The expression pattern of the immune responsive genes is presented as a heatmap (Figure 4C). It showed that American elm genotypic variations in responses to *Onu* were attributed mainly to genes encoding RPM1 disease resistance protein, phospholipase D beta 1, pathogenesis-related PR4, and thaumatin-like (PR5) proteins. These genes, except the phospholipase D beta 1, were more upregulated in the resistant than the susceptible genotype at 96 hpi (Figure 4C).

### 3.8. Expression Patterns of the DEGs Involved in Photosynthesis

**Figure 5:**
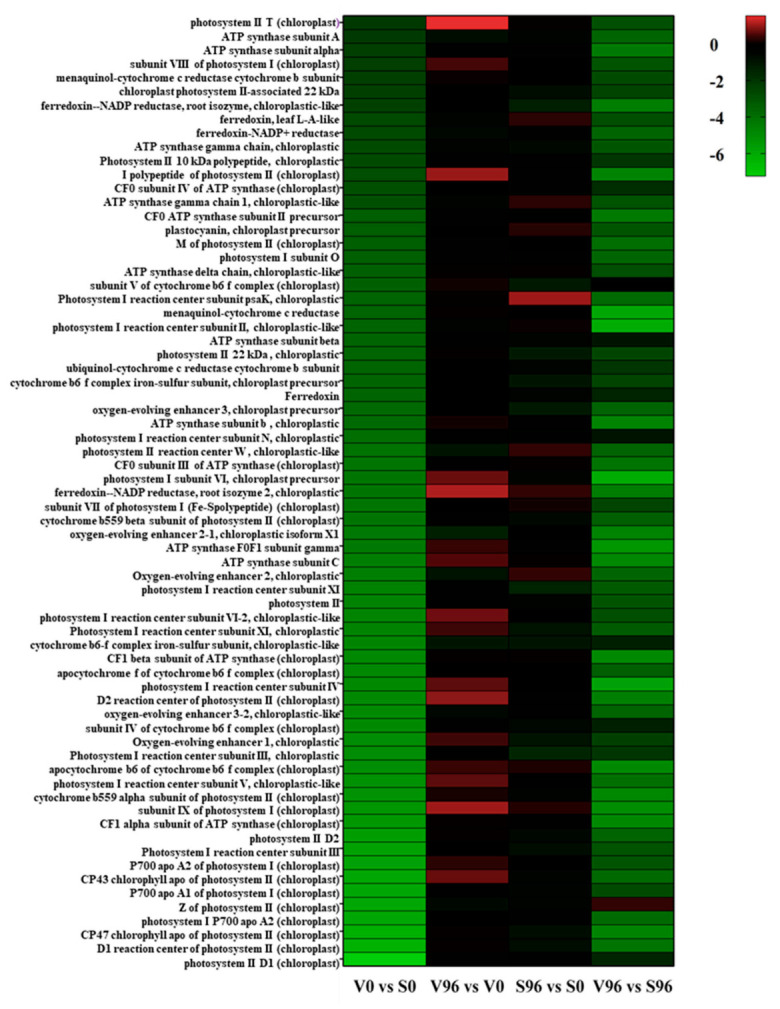
Heatmap of the significantly differentially expressed genes (DEGs) involved in photosynthesis in resistant (V) and susceptible (S) American elm genotypes at 0 and 96 h post-inoculation (hpi) with *Ophiostoma novo-ulmi*.

KEGG pathway enrichment analysis showed that photosynthesis was the most enriched pathway between the resistant and susceptible American elm genotypes at 0 hpi (Figure 5). Among the genes involved in the photosynthesis pathway, 69 genes were significantly differentially expressed, and all of them were downregulated in the resistant compared to the susceptible genotype at 0 hpi with *Onu*. Both photosystem I and photosystem II genes were significantly downregulated, and the most downregulated gene was photosystem II D1 (log2FC, −7.24). No significant photosynthesis-related DEGs were found in resistant or susceptible genotypes at 96 hpi.

### 3.9. Expression Profile of the DEGs Involved in the Biosynthesis of Phenylpropanoids

**Figure 6:**
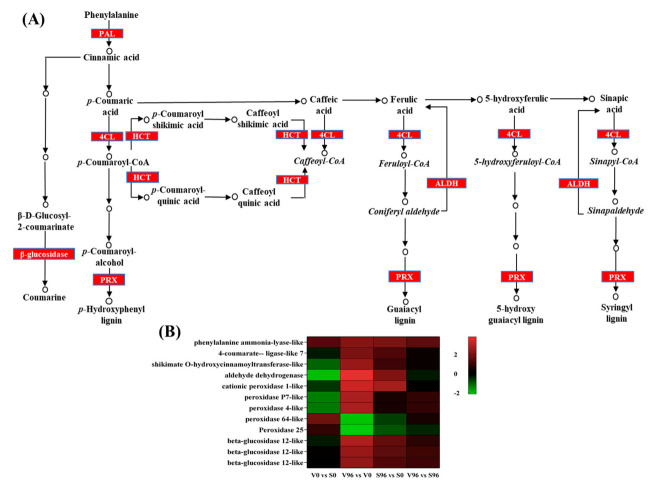
The expression pattern of genes coding for phenylpropanoid biosynthesis-related enzymes in *Ulmus americana* - *Ophiostoma novo-ulmi* interaction. (**A**) Phenylpropanoid biosynthesis pathway (KEGG database) highlighted significant differentially expressed genes (DEGs). (**B**) Expression profiles of the phenylpropanoid biosynthesis-related significantly differentially expressed genes in DED-resistant (V) and -susceptible (S) American elm genotypes.

The biosynthesis of secondary metabolites, specifically phenylpropanoids biosynthesis (Figure 6A), was the most *Onu-*responsive pathway enriched in both resistant and susceptible genotypes. Among the significant DEGs in resistant American elm-*Onu* pathosystem, ten genes involved in the phenylpropanoids biosynthesis pathway were significantly upregulated, whereas two were downregulated (Figure 6B). Five gene homologs of peroxidase and three of beta-glucosidase were identified among the significant DEGs. Three of the peroxidases showed similar expression profiles in both genotypes, which were upregulated in responses to the pathogen, and the other two were downregulated in the resistant genotype. Transcripts of the *aldehyde dehydrogenase*, *peroxidase P7-like,* and *peroxidase 4 like* genes showed higher log2 fold-change (3.80, 2.70, and 2.67, respectively) in the resistant compared to the susceptible genotype (2.10, 0.50, and 0.47, respectively) in response to *Onu*.

### 3.10. Validation of RNA-Seq Results by qRT-PCR

**Figure 7:**
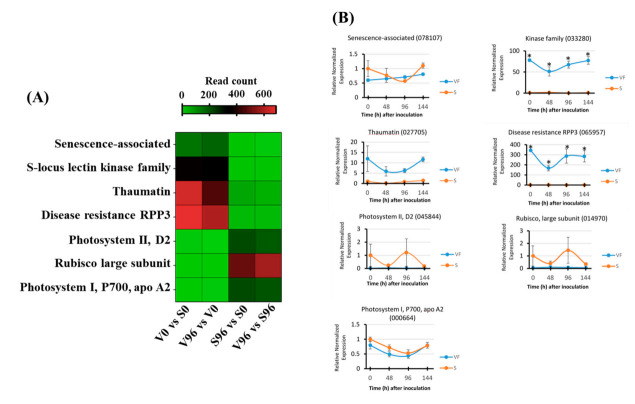
Validation by qRT-PCR of candidate genes identified by RNA-Seq analysis. (**A**) Expression of candidate genes via RNA-Seq in Valley Forge (V) and Susceptible (S) American elm genotypes at 0 and 96 hpi with *Ophiostoma novo-ulmi*. (**B**) Relative normalized expression of candidate genes for disease resistance and susceptibility in Valley Forge (VF) and Susceptible (S) genotypes quantified via qRT-PCR at 0, 48, 96, and 144 h post-inoculation (hpi). Asterisks (*) represent significant differences between VF and S within post-inoculation time points at *p* < 0.05, according to Tukey’s HSD test.

From the most significant DEGs between V96 and S96 samples, a representative subset of putative disease-related candidate genes was selected for validation of their expression patterns via qRT-PCR analysis. Following primer efficiency testing, only the primer pairs designed to amplify four upregulated genes (*senescence-associated* (078107), *kinase* family (033280), *thaumatin* (027705), and *disease resistance RPP3* (065957)), and three downregulated (*photosystem II D2* (045844), *rubisco large subunit* (014970), and *photosystem I P700 apo A2* (000664)) candidate genes were used for the validation. The gene expression analysis was performed using cDNA templates prepared from ‘Valley Forge’ and ‘Susceptible’ American elm mRNAs that were isolated at 0, 48, 96, and 144 h post-inoculation (Figure 7). For comparative purposes, a heatmap was generated with the read count of these genes obtained from the RNA-Seq analysis (Figure 7A). The relative normalized expression patterns of three upregulated genes, *thaumatin* (027705), *kinase* family (033280), and *disease resistance RPP3* (065957), matched their expression profiles measured via RNA-Seq, although only the *kinase* family (033280) and *disease resistance RPP3* (065957) genes were significantly upregulated in Valley Forge relative to susceptible at each time point (Figure 7B). The highest relative normalized expression among the upregulated candidate genes was detected for disease resistance RPP3 at V0 and V96 relative to S0 and S96 counterparts, which coincided with the expression of this gene in the RNA-Seq data (Figure 7A). Thaumatin and a kinase family gene also displayed comparable gene expression levels between the RNA-Seq data and the qPCR validation approach. In contrast, the expression patterns of a senescence-associated gene behaved differently between the RNA-Seq and the qRT-PCR analysis. While the senescence-associated gene was classified as upregulated in V0 and V96 relative to S0 and S96 samples in the RNA-Seq-based differential gene expression analysis, it appears to be downregulated in V0 relative to S0 samples, when its expression was measured via qRT-PCR (Figure 7B). Furthermore, the downregulated genes photosystem II D2, Rubisco large subunit, and photosystem I P700 apo A2 were also found to be downregulated in the qRT-PCR gene expression analysis, although there was no statistical evidence that they were differentially expressed.

## 4. Discussion

The highly destructive Dutch elm disease (DED) caused by the fungus *Optihostoma novo-ulmi* poses a significant threat to American elm [1,2,9]. Knowledge of the molecular mechanisms underlying the host–pathogen interactions in this pathosystem is, at best, fragmentary. Previously, transcriptome level changes in American elm calli during compatible interaction with *Onu* have been reported based on the analysis of a few hundred Expressed Sequence Tags (ESTs) [16]. A subsequent study in which susceptible and resistant lines of *U. americana* were inoculated with *O. novo-ulmi* allowed a comparison of molecular responses associated with compatible and incompatible interactions, respectively, but was limited to a small subset of disease-responsive genes that were assayed by qRT-PCR [9]. Therefore, the work reported herein aimed to obtain a comprehensive, genome-wide view of transcriptomic changes in resistant and susceptible genotypes of *U. americana* in response to DED. The plants of American elm genotypes with contrasting resistance to DED were inoculated with yeast spores of *Onu*, which germinated within the stem, where hyphae grew and spread through xylem vessels, inducing their cavitational embolism [30,31].

In this transcriptomics study, the BLASTX comparison yielded 55,499 unique genes in *U. americana*, which was approximately 2-fold higher than the number of unique genes in the *U. minor* transcriptome (Figure 2A). Previously, only 314 unisequences were isolated from *U. americana* calli inoculated with *Onu* [16]. The higher number of *U. minor* genes aligned to *U. americana* local DB and the higher number of unique genes in the *U. americana* transcriptome may be due to the sequencing platform used and the number of reads generated for each transcriptome. The *U. minor* transcriptome was sequenced in the 454 GS-FLX Titanium System, generating a total of 971,002 raw reads, whereas *U. americana* was sequenced in the Illumina HiSeq 2500 platform, generating an average of raw reads ranging from 66.4 to 75.8 million. In addition, it is well known that the Illumina HiSeq 2500 platform produces significantly higher sequence coverage than the 454 GS-FLX Titanium System [29,32]. Using the BLASTX software, the unique genes for each transcriptome were compared to the non-redundant protein database downloaded from NCBI, annotating approximately 61% genes from *U. minor* and 42% genes from *U. americana* as plant-related transcripts (Figure 2A). The remaining transcripts were annotated with functions from organisms belonging to other kingdoms, indicating the presence of plant endophyte genes that can be characterized in future studies. The lower number of plant-related transcripts in *U. americana* contrasted with the higher number of unique genes in this species relative to *U. minor* transcriptome suggests that the use of the Illumina 2500 platform could have generated a more diverse transcriptome of *U. americana*, which may include related endophytes. The transcriptome reported in this study represents the largest molecular source of information from *U. americana* obtained so far and would also be very helpful for annotating the *U. americana* genome that is publicly available in the NCBI database as an unassembled genome.

The transcriptomic analysis yielded a significant number of differentially expressed genes (DEGs) (Figure 3). The functional enrichment analysis of these DEGs indicated that the cellular and primary metabolic processes were the most highly enriched GO terms, wherease photosynthesis, carbon metabolism, and citrate cycle were the most enriched KEGG pathways between resistant ‘Valley Forge’ and susceptible elm genotypes regardless of the *Onu* inoculation (Table 2 and Table 3). The expression profile of the DEGs involved in the photosynthesis was significantly downregulated in the resistant American elm genotype compared to the susceptible genotype (Figure 5). This suggests that the genotypic variation mainly exists in the primary metabolism, and that enhanced primarily metabolic processes, especially photosynthesis, could be linked to the susceptiblity to DED [11]. In fact, the trade-off between growth and defense has previously been suggested as a defense mechanism against DED in the English elm-*Onu* pathosystem [33]. In that system, English elm trees compromise primary metabolic function at the expense of activating secondary metabolic processes for a successful plant defense [33,34,35]. GO terms of the *Onu*-responsive DEGs were enriched in response to stimulus, stress response, secondary metabolic process, and defense response in both the resistant and susceptible elm genotypes (Table 2).

Plants counteract pathogen attacks by activating a complex immune system. The primary immune response is known as pattern-triggered immunity (PTI), which is initiated by the perception of pathogen-associated molecular patterns (PAMPs) by pattern recognition receptors (PRRs) [36,37]. The second layer of defense, known as effector-triggered immunity (ETI), is initiated by plant resistance genes (R-genes) in response to pathogen effector proteins [38]. R-genes are usually characterized by a nucleotide-binding domain and a leucine-rich-repeat domain (NLR). NLRs stimulate rapid yet long-lasting defense responses including hypersensitive response followed by the production of reactive oxygen species (ROS), cell wall rigidification, synthesis of phytoalexins, hormonal signaling, and eventually the induction of PR genes [39,40] for suppressing the growth of invading pathogens. In general, resistance against biotrophic pathogens is regulated by the SA pathway, whereas jasmonic acid/ethylene (JA/ET) pathways provide resistance to necrotrophic pathogens [41]. The coordinated actions of both SA and JA are required to combat the hemibiotrophic pathogens [18], where SA is activated at the early biotrophic phase followed by JA’s induction during the necrotrophic phase [42]. Pathogenesis-related (PR) genes are a diverse group of genes and are considered the signature genes of SA and JA pathways. For instance, increased expression of the PR1, PR2, and PR5 genes represents the SA signaling pathway [43]. In contrast, increased expression of PR3, PR4, and PR12 corresponds to the activation of the JA pathway [44,45]. RPM1 encoding an NLR receptor protein [46] was significantly upregulated in resistant American elm–*Onu* interaction. On the other hand, phospholipase d beta 1 (*PLDβ1*), a type of phospholipase d (PLD) previously characterized as a negative regulator of RPM1 [47], was highly expressed in the susceptible American elm genotype in response to *Onu* (Figure 4). Furthermore, ROS-mediated hypersensitive response (HR), which is an essential mechanism of the plant’s defense against biotrophic and hemibiotrophic pathogens, is also negatively regulated by PLDβ1–PA signaling [48,49,50]. PLDβ1 expression is repressed by salicylic acid (SA), and the mutation of PLDβ1 enhanced SA production and signaling pathways upon pathogen infection [49,50]. On the other hand, *PR4* and *PR5* (thaumatin 1) genes were significantly upregulated in the resistant genotype, suggesting that resistance to DED in *U. americana* could be attributed to the simultaneous activation of SA and JA signaling, which agrees with previous findings [9,17]. Hence, it may be inferred that in the American elm-*Onu* pathosystem, the RPM1-mediated activation of ETI and repression of the PLDβ1 might be crucial for the resistance, and that the *Onu* pathogen might induce the *PLDβ1* in the susceptible, leading to the disease development.

The KEGG enrichment analysis showed that phenylpropanoid biosynthesis was remarkably enriched among the secondary metabolic processes in resistant and susceptible genotypes in response to *Onu*, which is in agreement with previous studies [15,16]. To estimate the significance of the phenylpropanoids biosynthesis pathway in DED resistance, we further extracted the significant DEGs involved in that pathway (Figure 6B). The biosynthesis of phenylpropanoids starts with the conversion of cinnamic acid from phenylalanine by phenyl ammonia-lyase (PAL), leading to the formation of different forms of phenolics [51,52]. The genes *4-coumarate ligase* (*4CL*), and *shikimate O-hydroxycinnamoyl transferase* (*HCT*) encode the enzymes involved in the synthesis of caffeoyl Co-A, feruloyl Co-A, 5-hydroxyferuloyl Co-A, and sinapoyl Co-A that act as precursors in the biosynthesis of different lignins [53], and the expression of these genes was significantly upregulated in the resistant genotype compared to the susceptible one in response to *Onu*. After a series of deamination, hydroxylation, methylation, and reduction, lignin monomers are produced in the cytoplasm and transported to the apoplast [53,54]. Finally, lignin is generally polymerized with three main types of monolignols (p-coumaryl alcohol, H unit; coniferyl alcohol, G unit; and sinapyl alcohol, S unit) by peroxidase (POD) in the secondary cell wall [55,56]. Interestingly, transcripts for peroxidases showed contrasting accumulation patterns in the resistant genotype challenged with *Onu*. For instance, *peroxidase P*7 and *peroxidase* 4*-like* transcripts were upregulated, whereas *peroxidase* 64*-like* and *peroxidase* 25 were downregulated (Figure 6B). This indicates that the activation of a specific peroxidase might be crucial for the polymerization of lignin and resistance against DED. Further characterization of these peroxidases and determination of the roles played by particular lignin polymers in resistance to DED warrants further studies.

Phenolic metabolites can be found either in soluble form in the cells or esterified and/or etherified within the cell wall for enhancing the cell-wall cross-linking [52]. The induction of the cell-wall-bound phenolics, lignin, and peroxidase is characterized as a resistance response in Norway spruce trees naturally infected with fungal pathogen *Ascocalyx abietina* [51]. The plant pathology literature contains several suggestions as to a role for fungal cell-wall-degrading enzymes in pathogenesis [57,58], and DED is no exception. The more aggressive *Onu* secretes higher amounts of glycosidases and exo-glycanases than the less aggressive *O. ulmi* under laboratory conditions [59,60]. Therefore, cell-wall reinforcement by inducing the cross-linking of phenolics and polymerization of lignin might be an inducible resistance mechanism of the American elm against DED. The different forms of phenolics and peroxidases for lignin biosynthesis and their contributions to resistance against Dutch elm disease warrant further study.

## 5. Conclusions

In conclusion, the present study is the first to assess genome-wide transcriptomic changes in American elm during the in vivo colonization of *Onu* fungus. Our data revealed that prior to infection (at 0 hpi), DEGs associated with photosynthesis and primary cellular metabolism were highly enriched and upregulated in the susceptible genotype, supporting the previously suggested notion of trade-offs between growth and defense in the DED pathosystem. After infection (at 96 hpi), transcriptomic changes in the resistant genotype could be explained in the light of effector-triggered immunity (ETI) as manifested by the enhanced expression of RPM1, PR genes, and genes in the phenylpropanoids biosynthesis pathway and lignin polymerization. In addition to elucidating the significant molecular changes in compatible and incompatible interactions of American elm and *Onu*, the present study provides transcriptomic data that would assist in the assembly, annotation, and characterization of the American elm genome, which itself is a significant milestone toward developing DED-resistant elm germplasm through breeding and biotechnology.

## Figures and Tables

**Table 1:** Overview of raw, mapped, and unmapped reads in *Ulmus americana*.

	Valley Forge		Susceptible	
	0 h	96 h	0 h	96 h
**Total reads**	75,742,361 (±0.8%)	73,144,879 (±3.9%)	66,441,814 (±8.6%)	75,518,322 (±4.6%)
**Alignment (%)**	73.7 (±0.7%)	71.5 (±1.5%)	70.1 (±0.9%)	69.8 (±0.4%)

Data represent the mean ± percent of standard error (denoted in brackets) of three biological replicates.

**Table 2:** Results of gene ontology (GO) enrichment analysis of differentially expressed genes.

Treatment	GO ID	GO Description	*p*-Value	FDR
**V0 vs. S0**	GO:0008152	Metabolic process	2.3 × 10^−110^	1.1 × 10^−106^
	GO:0009987	Cellular process	4 × 10^−94^	1 × 10^−90^
	GO:0044237	Cellular metabolic process	4.5 × 10^−83^	7.5 × 10^−80^
	GO:0044238	Primary metabolic process	1.2 × 10^−55^	.5 × 10^−52^
	GO:0050896	Response to stimulus	4.4 × 10^−46^	4.4 × 10^−43^
**V96 vs. V0**	GO:0050896	Response to stimulus	4.5 × 10^−14^	4.20 × 10^−11^
	GO:0006950	Response to stress	2.4 × 10^−12^	1.10 × 10^−9^
	GO:0042221	Response to chemical stimulus	1.9 × 10^−11^	5.90 × 10^−9^
	GO:0019748	Secondary metabolic process	2.5 × 10^−7^	5.80 × 10^−5^
	GO:0006952	Defense response	7.1 × 10^−7^	1.3 × 10^−4^
**S96 vs. S0**	GO:0006950	Response to stress	7.5 × 10^−14^	9.2 × 10^−11^
	GO:0050896	Response to stimulus	6.2 × 10^−13^	3.8 × 10^−10^
	GO:0008152	Metabolic process	6.9 × 10^−12^	2.8 × 10^−9^
	GO:0019748	Secondary metabolic process	4.6 × 10^−10^	1.4 × 10^−7^
	GO:0009628	Response to abiotic stimulus	2.4 × 10^−8^	5.8 × 10^−6^
**V96 vs. S96**	GO:0008152	Metabolic process	3.50 × 10^−107^	1.50 × 10^−103^
	GO:0009987	Cellular process	3.10 × 10^−79^	6.40 × 10^−76^
	GO:0044237	Cellular metabolic process	7.00 × 10^−77^	9.70 × 10^−74^
	GO:0044238	Primary metabolic process	3.50 × 10^−53^	3.60 × 10^−50^
	GO:0019538	Protein metabolic process	1.10 × 10^−42^	7.80 × 10^−40^

**Table 3:** Results of Kyoto Encyclopedia of Genes and Genomes pathway (KEGG) enrichment analysis.

Treatment	Pathway ID	Pathway Name	*p*-Value	FDR
**V0 vs. S0**	ko00195	Photosynthesis	1.7 × 10^−19^	1.1 × 10^−17^
	ko01200	Carbon metabolism	8.4 × 10^−12^	2.1 × 10^−10^
	ko00630	Glyoxylate and dicarboxylate metabolism	1 × 10^−8^	1.8 × 10^−7^
	ko00020	Citrate cycle (TCA cycle)	9.2 × 10^−8^	1.5 × 10^−6^
	ko00620	Pyruvate metabolism	9.8 × 10^−6^	1.4 × 10^−4^
**V96 vs. V0**	ko09110	Biosynthesis of secondary metabolites	7.9 × 10^−5^	1.9 × 10^−3^
	ko00940	Phenylpropanoid biosynthesis	4.5 × 10^−4^	8.2 × 10^−3^
	ko00350	Tyrosine metabolism	5.4 × 10^−3^	1.1 × 10^−2^
	ko00270	Cysteine and methionine metabolism	8.4 × 10^−3^	1.9 × 10^−2^
	ko00130	Ubiquinone and other terpenoid-quinone biosynthesis	2.4 × 10^−2^	2.2 × 10^−2^
**S96 vs. S0**	ko00630	Glyoxylate and dicarboxylate metabolism	2.9 × 10^−4^	8.5 × 10^−3^
	ko09110	Biosynthesis of secondary metabolites	3.6 × 10^−4^	8.5 × 10^−3^
	ko00071	Fatty acid degradation	5.7 × 10^−4^	1.0 × 10^−2^
	ko00910	Nitrogen metabolism	6.6 × 10^−4^	1.0 × 10^−2^
	ko00940	Phenylpropanoid biosynthesis	3.1 × 10^−3^	4.1 × 10^−2^
**V96 vs. S96**	ko00195	Photosynthesis	1.8 × 10^−17^	1.1 × 10^−15^
	Ko01200	Carbon metabolism	1.4 × 10^−15^	5.8 × 10^−14^
	ko00190	Oxidative phosphorylation	1 × 10^−12^	2.6 × 10^−11^
	ko00020	Citrate cycle (TCA cycle)	7.3 × 10^−10^	1.5 × 10^−8^
	ko00630	Glyoxylate and dicarboxylate metabolism	3.5 × 10^−9^	5.4 × 10^−8^

## Data Availability

RNA-Seq experimental data corresponding to *U. americana* and *O. novo-ulmi* raw reads for each biological replicate were deposited at the NCBI Sequence Read Archive as Binary Alignment Files under the accession number SRP149721. The assembled transcriptome of *U. americana* is also available at NCBI SRA library as a FASTA file under the same accession number.

## Supplementary Materials

The following supporting information can be downloaded at https://www.mdpi.com/article/10.3390/jof8020120/s1, Table S1: qRT-PCR primers used in this study; Table S2: Complete list of genes expressed in resistant and susceptible (V0 vs. S0) American elm genotypes at 0 hpi of *Ophiostoma* *novo-ulmi*; Table S3: Complete list of genes expressed in comparison between 0 hpi and 96 hpi of *Ophiostoma* *novo-ulmi* in resistant (Valley Forge; V) American elm genotype; Table S4: Complete list of genes expressed in comparison between 0 hpi and 96 hpi of *Ophiostoma* *novo-ulmi* in susceptible (S) American elm genotype; Table S5: Complete list of genes expressed in resistant and susceptible (V96 vs. S96) American elm genotypes at 96 hpi of *Ophiostoma* *novo-ulmi*.
