# Supplementary material for: Deciphering the Genome-Wide Transcriptomic Changes during Interactions of Resistant and Susceptible Genotypes of American Elm with Ophiostoma novo-ulmi
Source: J Fungi (Basel). 2022 Jan 26;8(2):120. doi: 10.3390/jof8020120 (PMC8874831; doi:10.3390/jof8020120)
Supplement: Supplementary file 1 [file jof-08-00120-s001.zip › Table S1.pdf]

Table S1: qRT-PCR primers used in this study.

Gene Description	Gene Bank Accession Number	Forward and reverse sequence (5'-3')
Senescence-associated	Unigene_078107	ACCTGATGCGGTGATGAGT<br>GGACGGTCCTTGAAAATCC
S-locus lectin kinase family	Unigene_033280	CACTGTCGCAGCATGAAAC<br>CAGGGGCTAGAGCATCGTA
Thaumatin	Unigene_027705	CAATCTTCCCGTTTTCGTG<br>AGGGCACAAATCGTTCAAA
Disease resistance RPP3	Unigene_065957	GGCATCATCAACCTCTTGG<br>CTCGCCAGCTTATCCACAT
Photosystem II, D2	Unigene_045844	CGTCGTGATCGATTCGTTT<br>CCTGTAAGCCAGCCACCTA
Rubisco, large subunit	Unigene_014970	CGTGCCCTAAGATTGGAAG<br>TTGTCACGCTCAACCTGAA
Photosystem I P700, apo A2	Unigene_000664	GTTGCTCCAGGCAACAAAC<br>TCGACTGGAAGACCGAGAG
Splicing factor 3B-F	FC325436	TTCCCCCTGAAAGAGAGGA<br>CAACCAACCGGATTTTCAG
